# Acknowledgement to reviewers 1st November 2017 to 31st October 2018

**DOI:** 10.1111/irv.12625

**Published:** 2018-12-23

The Editor would like to express his thanks to the following reviewers who gave of their time and expertise to help in the review process during the period 1st November 2017 to 31st October 2018:

Abdirizak, Fatuma

Aiello, Allison

Al‐Tawfiq, Jaffar

Alfilali, Muhammad

Alqahtani, Amani

Althouse, Ben

Andrews, Nick

Aoki, Fred

Azziz‐Baumgartner, Eduardo

Babkin, Igor V.

Banik, Gouri

Beck, Charles

Berhane, Yohannes

Bialasiewicz, Seweryn

Biggerstaff, Matthew

Boelle, Pierre‐yves

Bonfante, Francesco

Cao, Bin

Castilla, Jesús

Chakraborty, Apurba

Chale Dzul, Juan

Chambers, Catharine

Checchia, Paul

Chowell, Gerardo

Cleary, Paul

Cook, Alex

Couoh May, Daniel

Cowling, Benjamin

Crepey, Pascal

Cromer, Deborah

Dabrera, Gavin

Dahlgren, Fredrick

Daniels, Rodney

Dapat, Clyde

Davis, C. Todd

Ducatez, Mariette

Edelman, Laurel

Elfeky, Hanaa

Elliot, Alex

Emukule, Gideon

Engan, Mette

Engelhardt, Othmar

Falsey, Ann

Feng, Luzhao

Feng, Shuo

Ferdinands, Jill

Fischer, William

Fitzner, Julia

French, Clare

Frobert, Emilie

Gaglani, Manjusha

Garten, Rebecca

Gauger, Phillip

Gonzales, Jose

Govorkova, Elena

Gray, Gregory

Grohmann, Gary

Gubareva, Larisa

Hall, Jeffrey

Healy, Nuala

Hemida, M. G.

Heraud, Jean‐Michel

Homaira, Nusrat

Horton, Katherine C.

Huckriede, Anke

Hughes, Gareth

Hui, David

Hungnes, Olav

Huo, Xiang

Huppert, Amit

Ison, Michael

Kamigaki, Taro

Kaneki, Masao

Katz, Mark

Khan, Md Arifuzzaman

Khatami, Ameneh

Khurana, Surender

Kimura, Hirokazu

Krammer, Florian

Kyriakis, Constantinos

Lackenby, Angie

Lamson, Daryl

Lapidus, Nathanael

Lau, Eric

Lee, Dong‐Hun

Li, Feng

Lim, Wei‐Shen

Lin, Ping‐Ting

Ma, Stefan

Ma, Wenjun

Målbak, Kåre

Maltezou, Helen

Manicassamy, Balaji

Martin, Emily

Martineau, Adrian R.

Martinez‐Sobrido, Luis

Maurer‐Stroh, Sebastian

Mazick, Anne

Mboko, Wadzi

McCarthy, Noel

McCauley, John

Memish, Ziad

Monto, Arnold

Moorthy, Mahesh

Mosnier, Anne

Mubareka, Samira

Munywoki, Patrick

Muscatello, David

Muthuri, Stella

Myles, Puja

Nair, Harish

Ng, Sophia

Nguyen, Hang

Nguyen, Long

Ning, Zhangyong

Nokes, David

Nolting, Jacqueline

Nowalk, Mary Patricia

Nsoesie, Elaine

Olsen, Sonja

Omer, Saad

Oshitani, Hitoshi

Pasick, John

Paulson, Jim

Peiris, Malik

Pelat, Camille

Penttinen, Pasi

Power, Rosalie

Prill, Mila M.

Pyrc, Krzysztof

Qiu, Jianming

Rahman, Mahmudur

Ramey, Andrew

Rampling, Tommy

Rath, Barbara

Resch, Bernard

Riley, Steven

Riphagen‐Dalhuisen, Josien

Riviello, Elizabeth

Roberts, Richard

Robertson, Chris

Rockman, Steve

Russell, Margaret

Saito, Reiko

Sarazin, Marianne

Sartaj, Muhammad

Sawchuk, Larry

Schaefer, Rejane

Scragg, Robert

Seo, Sang Heui

Shanks, G. Dennis

Shinjoh (Shinjo), Masayoshi

Simon, Gaelle

Skrede, Steinar

Smith, Gavin

Spackman, Erica

Sridhar, Saranya

Stallknecht, David

Sullivan, Sheena

Sweet, Clive

Szyszkowicz, Mieczyslaw

Takashita, Emi

Tambyah, Paul

Tang, Julian

Tapia, Lorena

Todkill, Dan

Tompkins, S. Mark

Tøndel, Camilla

Torremorell, Montserrat

Ushijima, Hiroshi

Uyeki, Timothy

Van Buynder, Paul

Vijaykrishna, Dhanasekaran

Vincent, Amy

von Wissmann, Beatrix

Wæhre, Torgun

Wagner, Ralf

Walls, Tony

Warburton, Fiona

Waris, Matti

Warren‐Gash, Charlotte

Watanabe, Shinji

Webb, Steven

Webby, Richard

Wiersma, Lidewij

Wood, John

Xiyan, Xu

Yang, Wan

Yoshida, Laymyint

Zambon, Maria

Zanin, Mark

Zaraket, Hassan

Zhou, Fan

